# Tunable SiC-Based Photocatalysts for Hydrogen Generation and Environmental Remediation

**DOI:** 10.3390/ijms27020774

**Published:** 2026-01-13

**Authors:** Dina Bakranova, David Nagel, Nurlan Bakranov, Farida Kapsalamova, Danil Boukhvalov

**Affiliations:** 1 School of Information Technology and Applied Mathematics, SDU University, Kaskelen 040900, Kazakhstan; 2 Department of Electrical Engineering, School of Engineering and Applied Science, George Washington University, Washington, DC 20052, USA; 3 Research Group altAir Nanolab LLP, Almaty 050000, Kazakhstan; 4 School of Materials Science and Green Technologies, Kazakh-British Technical University, Almaty 050000, Kazakhstan; 5 College of Science, Institute of Materials Physics and Chemistry, Nanjing Forestry University, Nanjing 210037, China

**Keywords:** silicon carbide, photocatalysis, water splitting, carbon dioxide (CO_2_) reduction, dye degradation, machine learning, environmental remediation, wastewater treatment

## Abstract

Silicon carbide (SiC) has emerged as a robust and tunable semiconductor for advanced photocatalytic applications. This review provides a comprehensive overview of recent progress in the development of SiC-based materials for environmental remediation and solar-driven hydrogen production. Key aspects discussed include morphological engineering, heterostructure design, doping strategies, and plasmonic enhancement. Emphasis is placed on structure–activity relationships, insights from density functional theory (DFT) and machine learning (ML) models, and synergistic effects in composite systems. This review concludes with a critical analysis of current challenges and future research directions, highlighting the potential of SiC implementation as a sustainable platform for next-generation photocatalytic technologies.

## 1. Introduction

**Figure 1:**
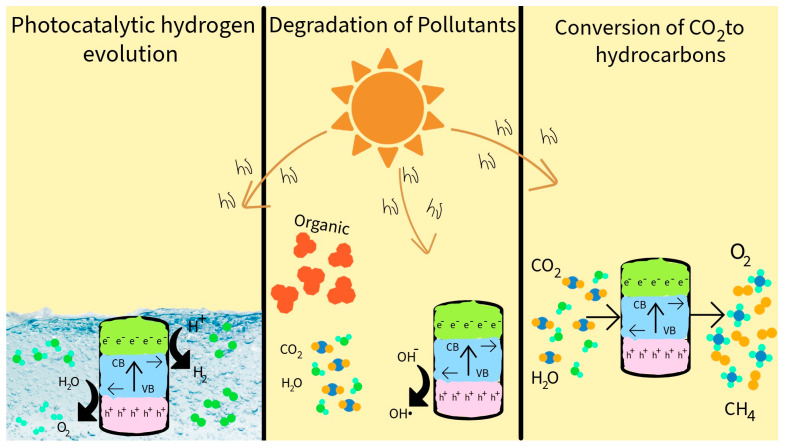
Schematic illustration of the photocatalytic applications of nanoparticles.

The increasing environmental burden resulting from industrial expansion, transportation, pharmaceutical consumption, and population growth has made environmental protection a global priority [1,2,3]. The adverse impacts of these developments manifest in climate change and growing contamination of air and water resources [4]. In response, photocatalytic technologies have attracted significant attention for their potential in environmental remediation and sustainable energy production [5,6,7,8], such as degradation of pharmaceutical residues, the photoreforming of alcohols, carbon dioxide (CO_2_) reduction, decomposition of nitrogen oxides (NO_x_), chlorofluorocarbons, solar-driven hydrogen production, water treatment, disinfection, and pollutant degradation [9,10,11,12,13,14,15,16,17] (Figure 1).

**Figure 2:**
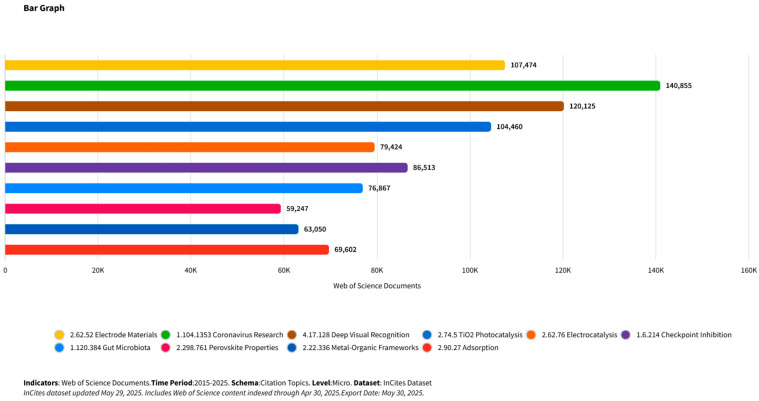
Chart of leading research topics by number of citations (based on InCites database data, 2025).

Among the most extensively studied photocatalysts (PCs), titanium dioxide (TiO_2_) dominates (Figure 2) due to its strong oxidative potential and stability. However, its wide band gap limits photoactivity to the ultraviolet (UV) region, which represents only a small fraction of solar radiation [18,19]. This limitation has motivated the search for alternative semiconductor materials and structural designs capable of harvesting a broader portion of the solar spectrum.

To address this limitation, various modification strategies, such as doping, heterostructure formation, and plasmonic enhancement, have been employed to broaden the optical response and improve charge separation [20,21,22,23,24]. An increasing number of materials are being developed as alternative PCs, including transition metal oxides, oxynitrides, sulfides, organic compounds, perovskites, and nanostructured composites [25,26,27,28,29,30,31,32,33,34,35,36,37]. Key requirements for such materials include suitable band edge positions, optical sensitivity, redox-active sites, and stability under illumination [38]. Moreover, minimizing exciton recombination and ensuring environmental safety and photostability are essential to long-term application [39]. However, many promising systems still suffer from instability, photocorrosion, or limited visible-light absorption, underscoring the need for more robust photocatalysts.

**Figure 3:**
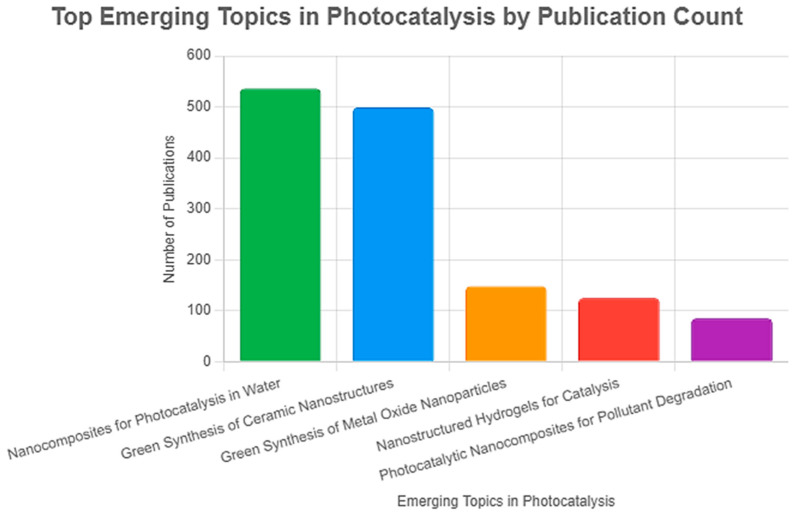
Top 5 Emerging Topics in photocatalysis (based on Research Horizon Navigator database data, 2025).

Among the wide range of materials suitable for photocatalysis based on their physicochemical properties, semiconductors stand out as the most extensively investigated class. Nanostructured and nanocomposite semiconductor materials have become central to current photocatalytic research. According to an analysis of Emerging Topics using the keyword “photocatalyst” in Research Horizon Navigator (based on Clarivate database data, 2025), “Nanocomposites for Photocatalytic and Antibacterial Applications in Water” was identified as a key Emerging Topic in 2025 (Figure 3). This trend reflects the increasing reliance on nanoscale engineering to manipulate carrier dynamics, light absorption, and interfacial reactions.

The application of nanotechnology in photocatalysis enables precise control over optical and electrical properties. Light absorption can be optimized by tuning scattering effects, while electronic behavior can be engineered through adjustments in diffusion length and charge-transport pathways. To suppress recombination of photogenerated carriers and enhance charge transfer, several strategies are employed, including semiconductor heterojunctions, incorporation of MXene phases, and the deposition of metal nanoparticles that form Schottky barriers [40,41]. These approaches illustrate a broader trend toward hierarchical and multifunctional photocatalyst architectures.

In addition to the extensively studied TiO_2_, other semiconductors, such as ZnO, WO_3_, Fe_2_O_3_, CdS, CuO, and their derivatives, have been widely explored as potential PCs. The synthesis toolbox for these materials includes both conventional techniques—such as hydrothermal synthesis, pyrolysis, chemical vapor deposition (CVD), and sol–gel methods—and more advanced approaches, such as 3D printing [42,43,44,45,46].

However, many of these semiconductor systems still face intrinsic limitations, such as poor visible-light absorption, rapid degradation, or environmental toxicity. This has prompted increasing interest in more durable, tunable, and environmentally benign materials that can maintain strong performance under operational conditions.

In recent years, silicon carbide (SiC) has emerged as a promising alternative photocatalyst owing to its high thermal and chemical stability, mechanical robustness, and tunable band gap (1.9–3.3 eV) depending on its polytype [47,48]. SiC is available in various morphologies and can be derived from sustainable resources such as agricultural waste, making it an attractive material for photocatalytic applications. These include water splitting, dye degradation, CO_2_ reduction, and wastewater treatment [47,49,50,51,52,53,54]. Unlike many oxide-based photocatalysts, SiC demonstrates exceptional resistance to photocorrosion and chemical degradation, enabling continuous operation under demanding conditions.

Hydrogen generation using SiC PCs has been demonstrated in both powder-based systems and photoelectrochemical (PEC) cells. While PEC approaches require improvements in charge separation and electrode durability [47,50,51,52,53], the synthesis of SiC powders is often simpler than the fabrication of stable coatings, as powders do not require substrate adhesion. SiC powders have been synthesized via a range of methods, including thermal plasma, carbothermal reduction, sol–gel, and pyrolysis [55,56,57,58,59,60]. Compared with thin-film fabrication, which requires precise control over film thickness, uniformity, and adhesion, powder-based SiC PCs offer a simpler and more scalable alternative. This makes them more suitable for large-scale applications, while reproducible thin-film photoabsorbers for PEC devices remain more difficult to manufacture [47,50,51,52,53].

The photocatalytic performance of SiC can be further enhanced through heterostructure formation, doping, and plasmonic effects. In parallel, the rapid development of density functional theory (DFT), time-dependent DFT, and machine learning-assisted simulations provides a deeper mechanistic understanding of electronic structure, defect energetics, and surface reactions in SiC. These tools are increasingly used to guide rational design of SiC-based photocatalysts and to predict performance trends that complement experimental observations [61,62,63,64,65,66].

This review aims to systematically summarize recent advances in SiC-based PCs, covering their structural, electronic, and morphological characteristics, as well as synthesis techniques and modification strategies. Particular emphasis is placed on computational modeling approaches (DFT and ML) that aid in understanding structure–activity relationships and optimizing material performance. By integrating experimental findings with theoretical insights, this review provides a comprehensive framework for understanding current capabilities, existing challenges, and future opportunities in the development of SiC for sustainable photocatalysis. This review concludes with a discussion of key challenges and future directions in the application of SiC for sustainable photocatalysis.

## 2. Crystal Structure and Polytypism of SiC

**Figure 4:**
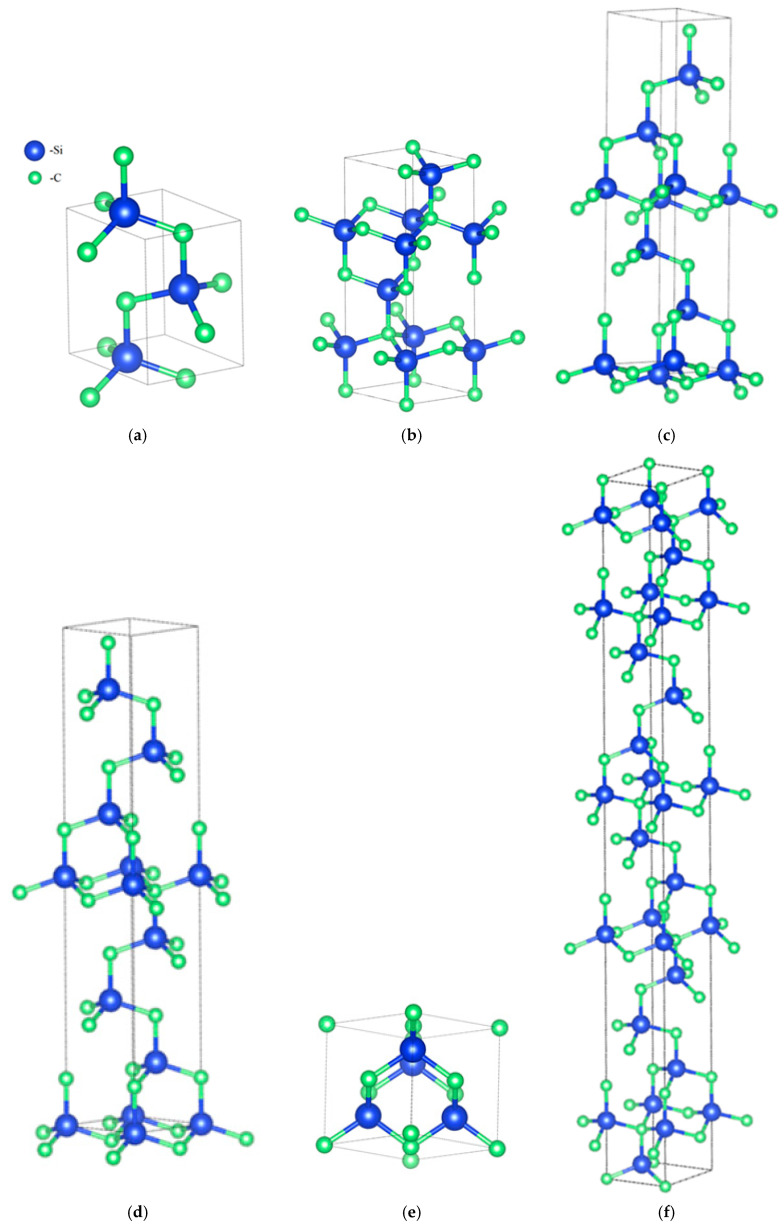
Crystal structures of SiC polytypes: (**a**) 2H SiC; (**b**) 4H-SiC; (**c**) 6H-SiC; (**d**) 8H SiC; (**e**) 3C-SiC; (**f**) 15R SiC.

SiC is a polymorphic semiconductor exhibiting a variety of crystalline structures known as polytypes. These structures differ in the stacking sequence of Si–C bilayers, resulting in variations in electronic and physical properties such as band gap, thermal conductivity, and crystal symmetry [67,68,69,70]. Over 250 SiC polytypes have been identified to date, with the most studied being cubic β-SiC (3C-SiC) and the hexagonal forms of α-SiC, including 4H-SiC and 6H-SiC (Figure 4).

The band gap of SiC varies significantly, from ~2.3 eV for 3C-SiC to >3.3 eV for 2H- and 4H-SiC, determining their spectral responsiveness. β-SiC can be activated under visible light, whereas α-SiC polytypes generally require UV excitation. The cubic 3C-SiC form can be synthesized at temperatures below 1700 °C, including renewable sources such as rice husks, providing an environmentally friendly route to nanostructured photocatalysts [71,72,73].

In contrast, 4H- and 6H-SiC require higher synthesis temperatures but offer superior thermal and mechanical stability. Their wider band gaps reduce visible-light activity, but mixed-phase systems combining α- and β-SiC can leverage the advantages of both. For example, in methane oxidative coupling, α-SiC provides structural integrity, while β-SiC improves selectivity and oxidation efficiency [74,75,76].

Overall, the choice of polytype depends on the reaction environment, light source, and the desired balance between optical activity and structural robustness.

## 3. Morphology-Controlled Synthesis of SiC Photocatalysts

The photocatalytic efficiency of SiC is strongly influenced by morphology, affecting surface area, light absorption, charge-carrier dynamics, and interfacial interactions. A wide range of synthetic methods enables SiC to be produced as nanowires, nanoparticles, films, porous foams, and composite structures, each optimized for specific photocatalytic applications.

### 3.1. One-Dimensional Structures

SiC nanowires provide high aspect ratio and effective charge-transport pathways, promoting enhanced carrier separation. Pyrolysis of polycarbosilane at ~1300 °C in a carbon-rich atmosphere is a well-established method for obtaining β-SiC nanowires with pronounced (111) orientation and high crystallinity [77]. Agricultural waste pyrolysis at >1500 °C yields rod-like β-SiC structures with surface areas of 19–64 m^2^/g [71].

Electrospinning followed by thermal treatment enables fabrication of CeO_2_/SiC nanofibers with high porosity, improving light harvesting and hydrogen evolution [78]. The hydrothermal deposition of CdS derivatives on β-SiC produces S-scheme heterojunctions such as ZnxCd_1−x_S/r-SiC, enhancing charge flow [79]. Noble-metal decoration (Pt and Au) further improves photocatalytic activity via plasmonic effects and hot-carrier injection [80,81].

### 3.2. Nanoparticles, Powders, and Biomass-Derived SiC

SiC powders are widely used because of their scalability and synthetic flexibility. Thermal plasma synthesis, microwave-assisted carbothermal reduction, and solid-state methods typically yield particles < 50 nm in size. Sol–gel processes enable precise control of nanoparticle size (30–50 nm) using silicon- and carbon-based precursors [55,56,57,58,59,60].

Laser ablation can generate metastable-phase SiC nanoparticles suitable for photocatalysis [82]. Comparable morphologies are found in SiC/C nanocomposites synthesized from agricultural residues. When modified with 5 wt% Pt, these show enhanced activity due to improved conductivity and suppressed recombination [83].

Biomass-derived SiC from rice husk pyrolysis provides porous, high-reactivity photocatalysts. Ball milling and hydroxylation enhance hydrophilicity and pollutant adsorption [71,84]. These materials show high performance in hydrogen evolution [85,86], dye degradation [87], and CO_2_ reduction [88].

Doping with elements such as Yb or Ce improves photocatalytic activity by introducing shallow trap states and enhancing charge separation [50,51,89]. The uniform loading of cocatalysts (Pt and Ni) increases catalytic efficiency by facilitating charge transfer and reducing recombination [81,90].

### 3.3. Porous Foams, Flakes, and Template-Assisted Architectures

Porous SiC foams and flakes exhibit interconnected pore structures enabling high mass transport and pollutant adsorption. Template-assisted sol–gel synthesis and dip-coating allow for the fabrication of SiC foams coated with TiO_2_, improving light utilization and scattering [91]. Surface hydroxylation increases hydrophilicity and reusability [92].

Electrochemically etched SiC flakes with exposed Si- or C-terminated faces show selective dye adsorption governed by Langmuir or Freundlich isotherms [93]. Waste derived silica–carbon precursors also enable low-cost, sustainable SiC production [94].

### 3.4. Hybrid and Composite SiC-Based Architectures

Hybrid systems integrating SiC with semiconductors, metals, or carbon materials show strong synergistic effects. Sol–gel synthesis yields Cr_2_O_3_–SiC–TiO_2_ [95] and Co_3_O_4_/SiC [96] composites with uniform nanophase dispersion and enhanced hydrogen evolution. Ternary structures such as Cu_2_O–SiC–g-C_3_N_4_ and MoS_2_–SiC–GO exploit favorable band alignments for efficient H_2_ and CO_2_ reduction [97].

**Figure 5:**
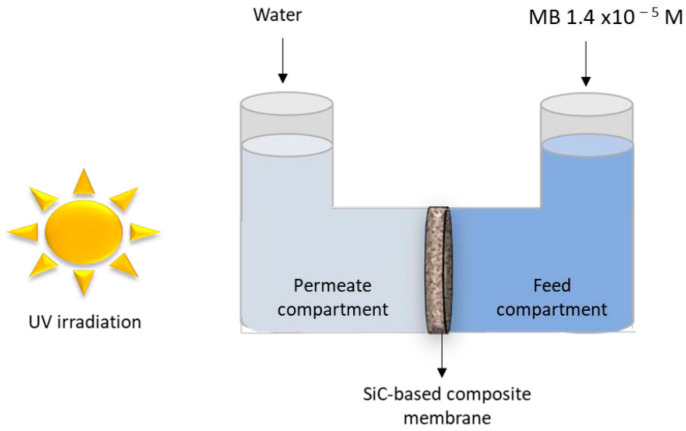
Schematic diagram of experimental diffusion setup for evaluating the performance of the SiC/α-Al_2_O_3_ composite material [98].

Composite membranes such as SiC/α-Al_2_O_3_ operate effectively in flow-through reactors, enabling selective pollutant removal (Figure 5) [98].

Other advanced morphologies, such as yolk–shell nanospheres (YSSC@TiO_2_) on SiC substrates, combine UV and visible-light responsiveness for multicolor dye degradation [99].

Microwave-assisted polymerization enables the formation of 3D-interconnected SiC/g-C_3_N_4_ frameworks. SiC whiskers in these composites act as heat centers, promoting urea polymerization and creating highly conductive networks that facilitate charge mobility and increase CO evolution rates [100].

### 3.5. Recycled and Waste-Derived SiC Photocatalysts

Recent research highlights the feasibility of synthesizing photocatalytically active SiC materials from industrial and agricultural waste. For example, composites derived from rice husk and modified with 5 wt% Pt exhibit improved hydrogen evolution due to enhanced charge mobility and reduced recombination [79,101].

Sonochemical methods offer an alternative activation route. Ultrasonic irradiation induces sonoluminescence, generating high-energy states that promote charge-carrier generation and hydroxyl radical formation in SiC nanowires [102].

**Figure 6:**
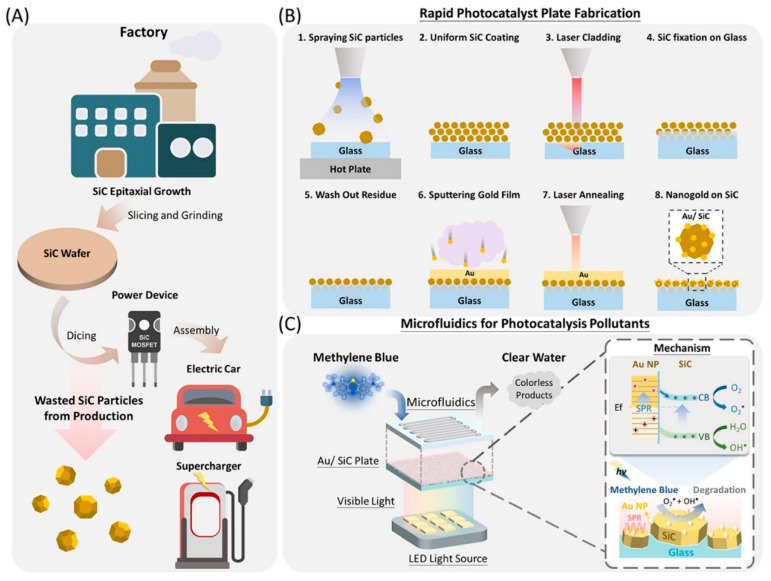
(**A**) SiC particles generated as byproducts within the power device production chain. (**B**) A rapid process for producing a photocatalytic plate with SiC. (**C**) Microfluidic device incorporating reconstituted SiC particles for the degradation of pollutants [103].

Recycled SiC particles from industrial waste have also been incorporated into microfluidic devices. Using infrared laser processing, these particles are immobilized on substrates and coated with Au films to form nanostructured photocatalytic plates. These devices achieve high methylene blue degradation rates and are compatible with scalable reactor designs (Figure 6) [103].

In addition to the above methods, SiC nanowires can also be fabricated from recycled silicon wafer waste, exhibiting significant photocurrent density (0.21 mA/cm^2^) and high conductivity under illumination [104]. Electrospinning [78] and catalytic chemical vapor deposition (CCVD) techniques yield fibrous SiC structures with high surface area and microwave activity, especially in ZrN_0.4_B_0.6_/SiC heterostructures [105].

Each SiC morphology offers distinct advantages tailored to specific photocatalytic applications. Nanowires and nanoparticles provide high surface-to-volume ratios and directional electron transport, while foams and flakes support scalable designs and enhanced pollutant accessibility. Hybrid composites expand functional versatility by enabling tunable band structures and synergistic charge interactions.

## 4. Modification Strategies for Enhancing the Photocatalytic Performance of SiC

While SiC possesses intrinsic advantages such as chemical inertness and a tunable band gap, its photocatalytic performance under visible light remains limited due to restricted visible-light absorption and fast recombination of photogenerated carriers, which significantly lower quantum efficiency. To overcome these drawbacks, various modification strategies have been developed, including heterostructure engineering, doping, and the incorporation of plasmonic components. These approaches aim not only to improve light harvesting but also to optimize charge migration pathways and suppress recombination through interfacial band engineering.

Constructing heterojunctions is one of the most effective ways to enhance the charge-carrier dynamics of SiC. For example, TiO_2_/SiC and SnO_2_/SiC composites combine the strong oxidizing power of metal oxides with the electron mobility and chemical stability of SiC [106,107,108]. In such systems, photogenerated electrons readily transfer from the oxide into SiC due to favorable band alignment, thereby prolonging carrier lifetime and accelerating surface redox reactions. Type II and Z-scheme heterostructures, such as those based on SiC–MoSe_2_ or SiC–PtSe_2_, facilitate efficient spatial separation of electrons and holes while simultaneously preserving strong redox potentials [109].

**Figure 7:**
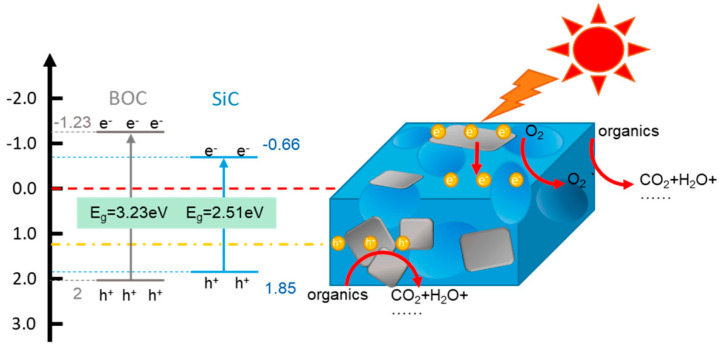
Proposed schematic of SiC-BOC composite PCs [112].

Two-dimensional semiconductors like BiOCl form favorable type I heterojunctions with SiC, where photoexcited electrons transfer from BiOCl to the conduction band of SiC, driving the formation of superoxide radicals (•O_2_^−^) [110,111]. Carbon-based coatings serve as conductive reservoirs that trap electrons, suppress recombination, and simultaneously increase pollutant affinity through π–π interactions [112] (Figure 7). In hydrothermally synthesized SiC/CdS composites, tight interfacial coupling enhances carrier lifetime and transport efficiency significantly compared with similar structures obtained by wet-chemical methods [113].

Doping SiC with suitable elements introduces intermediate energy states within the band gap, facilitating visible-light absorption and carrier trapping. Such dopant-derived states act as stepping stones that improve photon utilization while simultaneously tuning the Fermi level of the semiconductor. Non-metals such as B, N, P, and O have been incorporated into the SiC lattice through hydrothermal or sol–gel routes, enhancing both photocatalytic water splitting and dye degradation. For example, B-doped PMo_12_/SiC systems exhibit red-shifted absorption and extended carrier lifetimes, achieving 95.1% degradation of malachite green in 120 min [50].

Nitrogen doping increases hydrogen evolution to 205.3 μL/g·h—nearly double that of undoped SiC—due to improved carrier separation [114]. Type II heterostructures like SiS–SiC and SiS–P promote directional electron–hole migration across the interface, enabling water splitting and oxygen evolution at pH = 0 [115]. Alkaline-etched SiC (AE-SiC) features higher porosity and improves the dispersion and anchoring of ZIF-67 in peroxymonosulfate-based degradation of carbamazepine [116].

Metal doping also plays a crucial role. Ni-decorated β-SiC nanowires demonstrate high hydrogen evolution rates (3160.2 µmol/g·h) under visible light due to improved surface conductivity and reduced recombination [117]. Ce doping into H_2_Ti_2_O_5_/SiC reduces the band gap to 2.87 eV and increases the surface area, acting as efficient carrier traps [89]. In another example, atomic Yb doping in g-C_3_N_4_/SiC systems enables p–d orbital hybridization and π-backbonding with CO_2_, enhancing its selective reduction [51].

**Figure 8:**
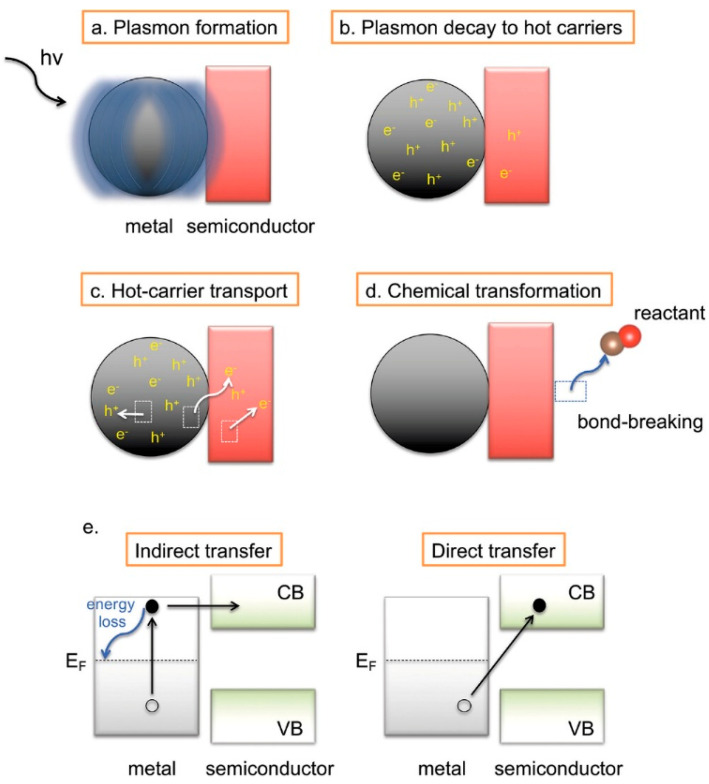
(**a**–**e**). Plasmonic excitation and hot-carrier transfer mechanisms at the interface of plasmonic metal nanoparticles (PM-NPs) and a semiconductor. (**a**–**d**) Schematic representations of various plasmon decay pathways, including non-radiative and radiative processes, resulting in the generation of hot carriers. (**e**) Illustration of direct and indirect hot-electron transfer from the PM-NP to the semiconductor, highlighting energy loss in the indirect pathway. EF, CB, and VB denote the Fermi level, conduction band, and valence band, respectively. Solid and hollow circles represent electrons and holes [120].

Incorporating plasmonic nanoparticles (e.g., Au, Ag, and Pd) introduces localized surface plasmon resonance (LSPR), which enhances light absorption and generates energetic “hot carriers” that can participate in redox reactions. These hot carriers improve catalytic turnover by injecting high-energy electrons directly into SiC or by amplifying local electromagnetic fields that boost excitation probabilities. These effects are both thermal (local heating) and non-thermal (carrier injection), though the latter is more desirable for enabling alternative mechanistic pathways and lowering reaction barriers [118,119]. The behavior of plasmonic PCs depends on particle size and morphology. Small (<20 nm) nanoparticles favor hot-carrier generation, while larger ones (>70 nm) predominantly scatter light (Figure 8a–e) [120].

At the interface between plasmonic metals and SiC, a Schottky barrier forms, facilitating hot electron transfer into the semiconductor and enabling charge-driven reactions. Metal decoration strongly affects photocatalytic performance. Noble metals act as electron mediators that accelerate interfacial charge extraction and reduce recombination [121]. For example, Pt addition to SiC and graphene enhances hydrogen generation by a factor of 175, with a rate of 2980 µmol·g^−1^·h^−1^ due to synergistic interactions in β-SiC/graphene/Pt structures [122]. Ag nanoparticles extend light absorption up to 463 nm, raising AQE to 7.3% at 420 nm in Ag/SiC/g-C_3_N_4_ composites. The incorporation of reduced graphene oxide (RGO) into SiC or GaN systems enhances conductivity and charge separation. Band gaps are reduced to 2.74 eV for SiC/RGO and 2.5 eV for GaN/RGO, extending visible-light absorption and improving water-splitting performance [123,124,125].

**Figure 9:**
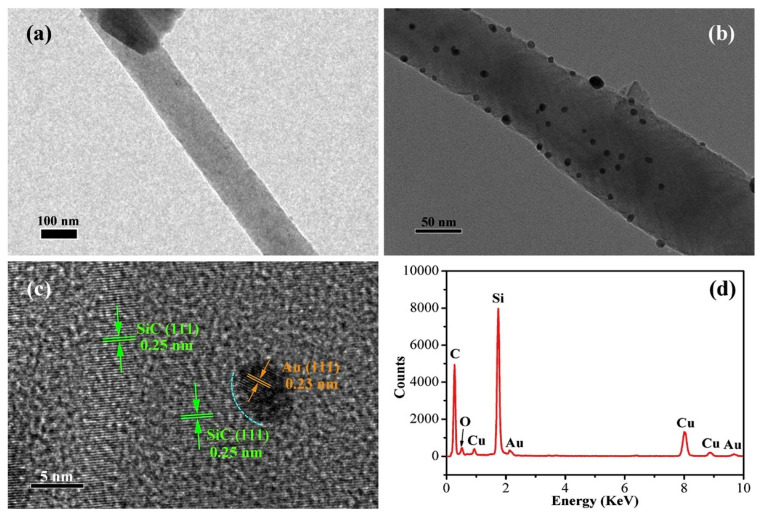
Typical TEM images of (**a**) SiC NWs and (**b**) Au/SiC-0.5 wt% nanocomposites, (**c**) corresponding HRTEM image of the 0.5 wt% Au NPs anchored on the SiC NWs, and (**d**) EDS spectrum of the Au/SiC-0.5 wt% nanocomposites (reproduced with permission, copyright Elsevier 2018 [80]).

Plasmonic nanoparticles such as Au, Ag, and Pd have been incorporated into SiC matrices to exploit localized surface plasmon resonance (LSPR). Upon light exposure, these structures generate hot carriers that activate SiC and trigger photocatalytic reactions [126,127]. In Au/SiC nanocomposites, 7–12 nm Au nanoparticles deposited on SiC nanowires form effective heterojunctions. The optimal loading (~0.5 wt%) maximizes photocatalytic activity by balancing light absorption and avoiding excessive recombination caused by dense metal coverage (Figure 9) [80].

Similarly, in Ag@AgCl–SiC systems, SPR-driven electron excitation enhances •OH radical generation, improving degradation efficiency in photo-Fenton-like processes [128]. Other systems, such as Ga/graphene/SiC, extend light absorption into the near-infrared and simultaneously enable charge delocalization via graphene, which strengthens both catalytic and spectroscopic responses [129].

Ternary composites that combine SiC with plasmonic metals and secondary semiconductors often outperform binary systems. For example, Ag/SiC/g-C_3_N_4_ exhibits a hydrogen evolution rate of 2971 µmol/g/h and an apparent quantum efficiency (AQE) of 7.3% at 420 nm—about nine times higher than that of bare g-C_3_N_4_ [130]. MoS_2_/SiC/GO structures further improve charge transport through graphene oxide, while MoS_2_ provides active catalytic sites, achieving quantum yields of up to 21.69% [131].

The superior performance of these systems results from multiple synergistic mechanisms: extended light absorption, directional charge separation, suppression of recombination, and increased density of reactive sites. Such cooperative interactions are essential to maximizing photonic and catalytic efficiency in SiC-based photocatalysts. SnO_2_@SiC composites outperform individual components by up to 3× in hydrogen evolution, with stable operation over 14 h [132]. Other heterostructures, such as Al_2_CO/SiC [133], β-AsP/SiC [134], GaN/SiC [125], MoSSe/SiC, and WSSe/SiC [135], promote charge-transfer and reaction kinetics. Combinations such as doping + plasmonic enhancement or heterostructuring + carbon coating consistently yield performance gains exceeding those achieved by any single-modification strategy.

In summary, the modification of SiC through heterojunction formation, targeted doping, and plasmonic enhancement significantly broadens its photocatalytic utility. These synergistic approaches collectively tune the electronic structure, expand visible-light response, and optimize interfacial charge separation, providing a rational pathway toward high-efficiency SiC-based photocatalysts. The integration of complementary modifications into rationally designed architecture continues to drive advances in SiC-based photocatalysis.

## 5. Computational and Theoretical Modeling of SiC Photocatalysts

Computational modeling plays a critical role in understanding and optimizing the photocatalytic behavior of SiC materials. Techniques such as density functional theory (DFT), molecular dynamics (MD), kinetic modeling, thermodynamic analysis, and machine learning (ML) enable detailed investigation of electronic structure, surface reactivity, charge-transfer processes, and reaction energetics. Their integration with experiments accelerates material discovery and supports rational design of high-performance SiC photocatalysts.

It should be noted that the calculated electronic properties of SiC strongly depend on the choice of the exchange-correlation functional. Semi-local functionals such as PBE systematically underestimate the band gap of SiC polytypes (e.g., 3C-, 4H-, and 6H-SiC), whereas hybrid functionals (e.g., HSE06) and many-body perturbation approaches (e.g., GW) provide values in closer agreement with experimental data. For example, while PBE typically predicts band gaps below 2.0 eV for 3C-SiC, HSE06 and GW calculations yield values in the range of 2.3–2.4 eV, consistent with optical measurements. This discrepancy must be considered when interpreting theoretical predictions related to light absorption, defect levels, and charge-transfer energetics in SiC-based photocatalysts.

DFT calculations are widely employed to investigate band structures, heterojunction properties, and reaction energetics. In SiC/PtSe_2_ and CrSSe/SiC Z-scheme systems, first-principles simulations reveal indirect band gaps of 1.52 eV and 1.14 eV, respectively, along with strong internal electric fields that promote directional charge flow. These findings predict solar-to-hydrogen (STH) efficiency of up to 42.2% [136,137]. In 2D BiOCl/SiC heterostructures, DFT indicates reduced activation barriers for CO_2_-to-CO conversion due to interface-induced charge redistribution [138]. For strained tetragonal SiC (t-SiC), HSE06 hybrid functionals predict band gap narrowing under biaxial tensile strain, improving visible-light absorption while maintaining structural stability [47]. DFT studies of Yb-doped SiC demonstrate p–d orbital hybridization that lowers the energy barrier for *COO formation, increasing CO evolution rates more than tenfold [51]. While advanced approaches such as GW and GW-BSE offer superior accuracy for band gap and excitonic effects, their computational cost scales unfavorably with system size, limiting their applicability to idealized models or small unit cells. In contrast, hybrid DFT methods (e.g., HSE06) represent a practical compromise between accuracy and computational feasibility for modeling extended surfaces, defect-rich structures, and heterojunction interfaces relevant to realistic SiC photocatalysts [139,140].

Molecular dynamics (MD) simulations, especially when combined with experimental techniques like X-ray photoelectron spectroscopy (XPS), provide information on surface reactivity and oxidation behavior. For instance, RS-SiC in contact with TiO_2_ undergoes hydroxyl radical-assisted surface oxidation, while its porous structure enables enhanced accessibility to active sites [141]. Kinetic models based on pseudo-first-order reactions have been used to quantify pollutant degradation on defect-rich SiC (dSC) surfaces, with rate constants of 3.62 × 10^−3^ and 4.09 × 10^−3^ s^−1^ for dichlorvos and azoxystrobin, respectively [53]. Thermodynamic simulations of methylene blue adsorption indicate that the Si-terminated face conforms to the Langmuir model, whereas the C-terminated face follows a dual Langmuir–Freundlich model, reflecting differences in polarity and surface roughness [93].

ML enables the prediction of SiC properties across compositional, structural, and process-related variables. Models such as support vector regression (SVR), random forest (RFR), and extreme learning machine (ELM) accurately estimate thermal conductivity, with ELM showing substantial reductions in MAE (−40.5%) and RMSE (−24.4%) relative to baseline regressors. In band structure prediction [142], the DFTB–ML hybrid method reproduces electronic states near the Fermi level with high transferability [143]. ML-assisted defect modeling, such as for Si vacancies in 3C-SiC, captures charge-state-dependent entropic contributions and provides improved predictions of local electronic behavior [144].

Large-scale screening using ML-DFT integration has identified novel monolayer SiC derivatives suitable for photocatalytic water splitting, with some candidates showing carrier mobilities exceeding 10^4^ cm^2^ V^−1^ s^−1^ and strong deep-UV absorption [145]. ML is also used to optimize synthesis processes. Extreme learning algorithms accurately predict deposition rates and precursor consumption during low-temperature CVD of SiC thin films [146]. The ACO-BPNN method further reduces the epitaxial growth error of 4H-SiC to 4.03% [65].

The modeling of defects, such as Si-vacancies, using ML identifies an activation energy for spin polarization (36 meV), which improves photon interactions [147]. In defect engineering, ML-derived force fields (MLFFs) enable simulation of Shockley-type defects in 4H-SiC, providing insights into their influence on optical absorption and charge mobility [61]. Universal semi-empirical models such as DPA-Semi offer fast predictions of electronic behavior across semiconductor families, including SiC [148].

Hybrid modeling approaches combine the strengths of DFT, MD, and ML. DFT-informed ML extracts structure–property correlations from large datasets, while MD–ML frameworks accelerate simulation of realistic charge-carrier dynamics. Despite rapid progress, challenges include model interpretability, data quality, and the need for experimental validation to confirm predictions. For instance, simulations in [145] demonstrated that ML reduces analysis time for large datasets, a feat unachievable by traditional methods. However, ML requires high-quality training data and experimental validation to confirm predictions [65].

In summary, computational tools have become indispensable for advancing SiC-based PCs. The integration of DFT, MD, and ML enables prediction and optimization of band structure, defect chemistry, interfacial reactivity, and reaction kinetics. These tools significantly accelerate discovery, reduce experimental workload, and support the rational design of high-efficiency SiC photocatalysts for hydrogen production, CO_2_ reduction, and pollutant degradation [148].

## 6. Water Splitting on SiC-Based Photocatalysts

Photocatalytic water splitting represents a sustainable approach to hydrogen production, driven by solar energy. SiC, owing to its chemical stability, tunable band gap, and morphological versatility, has demonstrated considerable potential in this field. Through careful design of heterostructures, surface modifications, and composite architectures, SiC-based systems can achieve high activity under both UV and visible light. These strategies are particularly important for compensating the limited intrinsic visible-light absorption and rapid charge-carrier recombination of pristine SiC.

Among various architectures, Z-scheme heterojunctions have proven particularly effective, as they retain high redox potential while enabling efficient spatial separation of charge carriers. In SiC/PtSe_2_ systems, electrons from the conduction band (CB) of PtSe_2_ recombine with holes in the valence band (VB) of SiC, leaving photogenerated electrons in SiC and holes in PtSe_2_ available for hydrogen and oxygen evolution, respectively. This configuration has achieved a theoretical STH efficiency of 42.2% under simulated solar irradiation [136,137]. Such high predicted STH values underline the importance of interfacial band alignment for overall device performance.

Other layered heterostructures, such as BiOCl/SiC and SiC–g-C_3_N_4_, leverage 2D/2D interfaces to improve carrier mobility and increase surface reaction rates, as supported by DFT calculations and experimental photoactivity. The intimate contact between 2D layers shortens carrier transport distances and maximizes utilization of active sites.

From a theoretical perspective, the HER activity of SiC-based photocatalysts is primarily governed by the Gibbs free energy of hydrogen adsorption (${\Delta G}_{H}^{*}$), which serves as a universal descriptor of HER kinetics. Ideal HER catalysts exhibit ${\Delta G}_{H}^{*}$ values close to zero, indicating balanced hydrogen adsorption and desorption. DFT studies report that pristine SiC surfaces typically show relatively positive ${\Delta G}_{H}^{*}$ values, reflecting weak hydrogen binding and sluggish HER kinetics. In contrast, defect-engineered SiC and heterojunction interfaces significantly reduce ${\Delta G}_{H}^{*}$, bringing it closer to thermoneutral conditions [149,150,151,152].

The introduction of oxygen vacancies or structural defects can further enhance photocatalytic activity by providing trap states that extend carrier lifetimes. In CeO_2_/SiC nanofibers, oxygen vacancies act as electron sinks, increasing hydrogen evolution to 5208 µmol/g under visible light [78]. Similarly, strained t-SiC (subjected to 8% biaxial tensile deformation) exhibits a reduced band gap and enhanced absorption, with predicted H_2_ generation rates exceeding 2.3 × 10^5^ µmol/g [47]. This highlights the utility of mechanical strain as a tool for band gap engineering and activity enhancement.

Morphological engineering—especially the formation of porous nanowire arrays—improves photocatalytic performance by increasing the surface area and creating continuous charge-transport pathways. These structures not only facilitate light absorption and charge separation but also enhance interaction with water molecules, leading to improved reaction kinetics. SiC/ZnO nanostructures have demonstrated efficient hydrogen evolution even without sacrificial agents, indicating the formation of a complete photocatalytic cycle [153,154].

Modification of SiC with noble metals such as Pt and Au can significantly improve photocatalytic activity by enhancing charge separation and promoting surface redox reactions. Pt-decorated SiC nanowires, for example, exhibit hydrogen production rates of 4572 µL/g/h under Xe-lamp illumination, with Pt nanoparticles (~2.5 nm) serving as efficient electron sinks [81]. However, excessive metal loading can result in light shielding and increased recombination. For Au-modified SiC systems, optimal loading (typically <1 wt%) is critical to balancing LSPR effects with overall light penetration [80,81,155]. Therefore, precise control over cocatalyst loading is essential to maximizing performance.

More complex PCs combining SiC with both plasmonic and semiconducting components have shown superior performance. Ag/SiC/g-C_3_N_4_ composites, for instance, exhibit an AQE of 7.3% at 420 nm and hydrogen evolution rates of 2971 µmol/g/h, significantly outperforming their individual components [130]. NiO_x_/SiC/CNOs composites demonstrate rates of 3160.2 µmol/g/h, attributed to reduced photoluminescence intensity and improved carrier separation [117]. These ternary systems benefit from synergistic effects among conductive, light-harvesting, and catalytic components.

The integration of graphene or graphene oxide (GO) further improves photocatalytic behavior. GO sheets facilitate fast electron transport, enhance light absorption, and suppress recombination. In MoS_2_/SiC/GO composites, the GO layers serve as electron mediators, while MoS_2_ provides active sites, resulting in quantum yields of up to 21.69% in the 400–700 nm range [131]. The presence of oxygenated groups on GO also supports uniform catalyst dispersion and high interfacial conductivity [156,157].

A comparative overview of SiC-based PCs for water splitting is provided in Table 1, Table 2 and Table 3. These tables summarize key parameters, including band gap, light source, electrolyte, structural features, and hydrogen evolution rates. Among the investigated materials, the SiC/PtSe_2_ heterostructure demonstrates the highest theoretical solar-to-hydrogen (STH) conversion efficiency. The CeO_2_/SiC system shows enhanced performance under visible-light irradiation, achieving superior hydrogen evolution rates. Meanwhile, the Ag/SiC/g-C_3_N_4_ composite provides well-balanced performance, combining favorable hydrogen production rate, apparent quantum efficiency (AQE), and operational stability.

**Figure 10:**
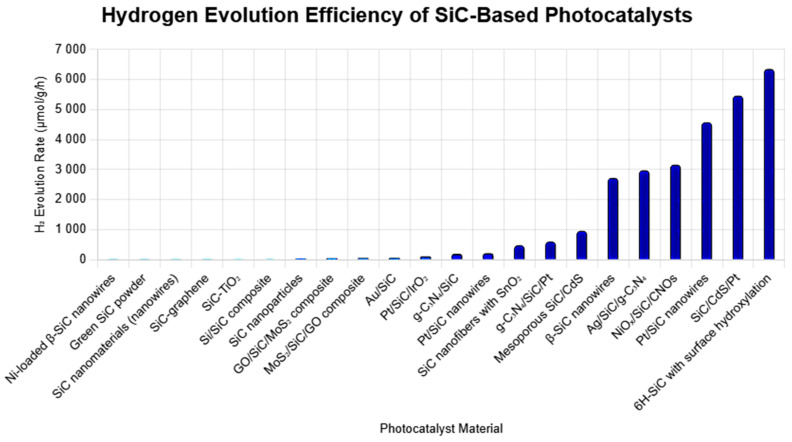
H_2_ evolution efficiency of SiC-based PCs.

The overall water-splitting performance of SiC-based PCs is determined by multiple factors, including electronic structure, interface engineering, surface area, and charge-transport pathways. Strategies such as Z-scheme design, defect engineering, and plasmonic enhancement, especially when combined, yield significant improvements in activity and stability. The ability of SiC to operate without sacrificial agents, tolerate harsh environments, and maintain structural integrity positions it as a strong candidate for integrated solar hydrogen production systems. Figure 10 represents the bar graph of H_2_ evolution efficiency of SiC-based PCs.

## 7. Photocatalytic Degradation of Organic Pollutants

The photocatalytic degradation of organic dyes and pollutants in wastewater is a critical environmental application, and SiC-based materials have shown considerable promise due to their stability, broad spectral responsiveness, and adaptability for composite design. Efficient dye degradation relies not only on light absorption but also on surface adsorption characteristics, generation of reactive oxygen species (ROS), and effective separation of charge carriers.

SiC and its composite PCs have garnered considerable attention for the degradation of organic contaminants in wastewater, including dyes, pharmaceuticals, and pesticides. These materials offer a unique combination of physicochemical stability, high surface area (in nanostructured forms), and the ability to form heterojunctions that enhance charge separation and reduce electron–hole recombination.

Upon illumination, SiC absorbs photons with energy greater than its band gap, generating electron–hole pairs (Equation (1)):

SiC + hν → e^−^ + h^+^ (1)

The photogenerated holes oxidize surface-bound water or hydroxide ions to form highly reactive hydroxyl radicals (Equations (2) and (3)):

h^+^ + H_2_O → •OH + H^+^ (2)

or

h^+^ + OH^−^ → •OH (3)

Meanwhile, conduction band electrons reduce dissolved oxygen to superoxide radicals (Equation (4)):

e^−^ + O_2_ → •O_2_^−^ (4)

Subsequent reactions yield other reactive oxygen species (ROS), such as hydroperoxyl (•HO_2_) and hydrogen peroxide (Equation (5)):

•O_2_^−^ + H^+^ → •HO_2_ •HO_2_ + •HO_2_ → H_2_O_2_ + O_2_ H_2_O_2_ + hν → 2•OH (5)

These ROS species oxidize organic pollutants, leading to their mineralization into CO_2_, H_2_O, and other benign products (Equation (6)):

C_x_H_y_N_z_O_w_ + •OH → CO_2_ + H_2_O + NO_3_^−^ + others (6)

Photocatalytic activity is often quantitatively assessed using kinetic models. Most dye degradation processes follow pseudo-first-order kinetics, represented by Equation (7):

ln(C_0_/C) = kt (7)

where C_0_ and C are the initial and residual pollutant concentrations at time t and k is the apparent rate constant. The linearity of the plot confirms the reaction order and allows for a comparison of different catalysts.

In many SiC-based systems, the presence of oxidants such as persulfate (S_2_O_8_^2−^) or sulfate (SO_4_^2−^) further enhances oxidative strength, enabling near-complete degradation of emerging contaminants [166]. In B-doped PMo_12_/SiC composites, a Z-type heterojunction promotes effective charge separation and extends the absorption edge. This system achieved 95.1% degradation of malachite green within 120 min, demonstrating that intentional doping and heterostructure formation can synergistically improve photocatalytic efficiency [50].

**Figure 11:**
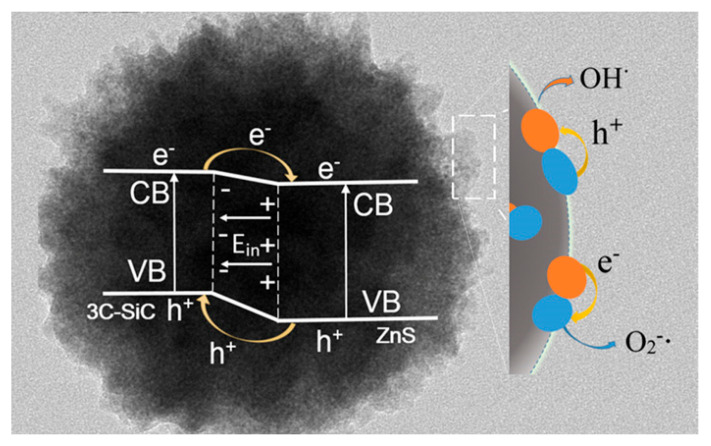
Illustration of the band gap and charge-transfer processes at the interface of 3C-SiC and ZnS nanocrystals [167].

The 3C-SiC/ZnS nanocomposite is a representative system utilizing directional charge transfer. Upon visible-light irradiation, photoexcited electrons migrate from SiC to ZnS, where they reduce O_2_ to O_2_^−^•. These intermediates subsequently generate H_2_O_2_ and •OH, while holes in SiC oxidize water or dye molecules directly. This p–n junction structure effectively suppresses recombination and enhances overall reactivity [167] (Figure 11).

**Figure 12:**
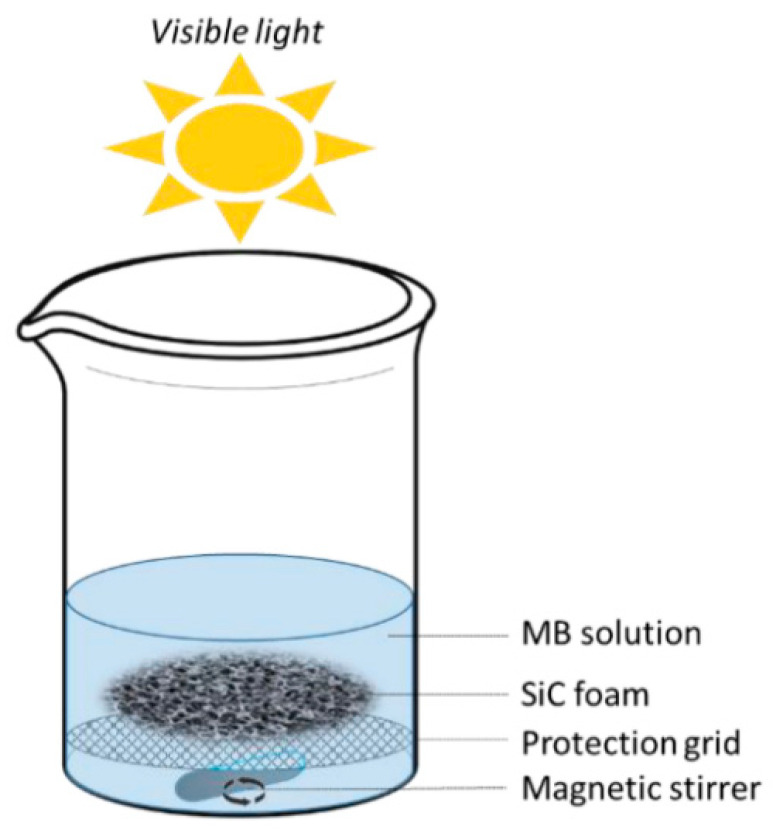
Setup used for the photocatalytic degradation of MB under visible light with SiC foam [92].

Structural morphology significantly influences photocatalytic behavior. SiC foams, for instance, can adsorb up to 26% of methylene blue (MB) in the dark through chemisorption, following pseudo-second-order kinetics (R^2^ = 0.9707). Under 150 W visible light, the degradation of MB increases to 88% within 8 h. Surface hydroxylation improves wettability and enhances both adsorption and light-induced activity [92] (Figure 12).

Porous SiC flakes, synthesized via electrochemical etching, exhibit selective adsorption of cationic dyes due to their negatively charged surfaces. Langmuir-type adsorption dominates on Si-terminated faces, while mixed Langmuir–Freundlich behavior is observed on C-terminated ones [93]. This pre-adsorption facilitates localized pollutant accumulation and accelerates degradation under illumination.

SiC/α-Al_2_O_3_ composite membranes used in diffusion cells exhibit selective permeability and dye degradation across a defined membrane area. Their integration into borosilicate reactors allows for controlled diffusion and efficient reaction at the interface. These systems enable scalable and continuous-flow photocatalysis under operational conditions relevant to industrial wastewater treatment [98].

SiC nanowires subjected to ultrasonic treatment exhibit enhanced degradation kinetics due to sonoluminescence-induced generation of •OH radicals. These systems outperform commercial TiO_2_ (P25), confirming the suitability of SiC for hybrid sonophotocatalytic applications [102]. In acoustic catalysis, SiC nanowires thus combine mechanical, optical, and chemical activation modes [102]. For wastewater treatment, SiC is effective through both radical generation and adsorption. For instance, Ag_2_MoO_4_/AgCl/SiC heterostructures enable 98.68% cefaclor degradation within 30 min using type II and S-scheme charge pathways [168].

Table 4 presents a comparative overview of the photocatalytic performance of various SiC-based systems, including degradation rates, light sources, and targeted dyes. Among the evaluated composites, SnO_2_/SiC demonstrates exceptional activity by achieving 99% degradation of methyl orange within 45 min under visible light. The Bi_2_WO_6_/SiC system enhances the degradation efficiency of Rhodamine B by a factor of 3.7 compared with pristine Bi_2_WO_6_. Additionally, Ag_2_CO_3_/SiC exhibits remarkable performance under natural sunlight, reaching 98% degradation of methylene blue.

These examples highlight the versatility and efficiency of SiC in both UV and visible-light-driven systems. A ternary MoS_2_/SiC/GO composite (SMG-2.5) achieved >90% RhB degradation in 60 min, with an AQY of 21.69% at 400–700 nm [169].

**Table 4:** Summary of performance of some SiC-based heterostructures for dye degradation.

Material	Light Source	Dye	Degradation Rate	Shape/Size	Source
SnO_2_/SiC	Visible light	Methyl orange (MO)	99% in 45 min	Nanosheets	[108]
Bi_2_WO_6_/SiC	Visible light	Rhodamine B	3.7 times higher than Bi_2_WO_6_	Petal microsphere	[170]
ZnO/SiC	UV light	Methylene blue	95.7% in 120 min	Rod-shaped, flower-like	[171]
TiO_2_/β-SiC foam	Not specified	Rhodamine B	~90%	Foam	[172]
Graphene-covered SiC powder (GCSP)	UV light	Rhodamine B	>100% enhancement over pristine SiC	Powder	[87]
TiO_2_/SiO_2_/SiC	UV light	Methylene blue	72%	Membrane	[173]
TiO_2_/SiC foam	UV light	Pyrimethanil	88%	Foam	[174]
Bi_2_WO_6_/SiC(O)	UV light	Rhodamine B	~90%	Nanoparticles in SiC (O) matrix	[175]
TiO_2_/β-SiC	UV–Vis (100 W)	Methylene blue and methyl orange	Higher for MB than methyl orange	Anatase TiO_2_ agglomerates	[176]
TiO_2_/Au-CNT on SiC	Solar light	Rhodamine B	~98.5%	Composite on SiC ceramic	[177]
YSSC@TiO_2_	UV and visible light	Methylene blue and Congo red	High for MB and Congo red	Yolk–shell nanospheres	[99]
SiC@SiO_2_ nanocapsules	Visible light	Methylene blue	~95% in 160 min	Hexagonal platelets (120–150 nm)	[178]
Cu_2_O-SiC/g-C_3_N_4_	Visible light	Methyl orange	93.70%	Ternary composite	[179]
Ag_2_CO_3_/SiC	Natural sunlight	Methylene blue	98%	Nanostructure	[180]

SiC-based PCs exhibit strong performance in dye degradation through a combination of light-induced ROS generation, selective adsorption, and improved charge-carrier separation. Morphological control, heterostructure engineering, and surface functionalization further enhance efficiency and stability. The ability to operate in flow-through systems and hybrid configurations makes SiC a promising platform for practical wastewater treatment technologies.

## 8. Photocatalytic CO_2_ Reduction on SiC-Based Systems

Photocatalytic reduction of carbon dioxide offers a dual benefit: mitigating greenhouse gas emissions and generating valuable chemical fuels, such as CO, CH_4_, and CH_3_OH. However, the process faces kinetic and thermodynamic limitations due to the inertness of CO_2_ and rapid recombination of photogenerated carriers. SiC-based materials, with their structural robustness and electronic tunability, present promising solutions through engineered heterostructures, composite systems, and interfacial design.

Photothermal strategies exploit localized heating under light irradiation to accelerate reaction kinetics. Granular SiC exposed to laser light has achieved 100% selectivity toward methanol, attributed to enhanced CO_2_ adsorption and desorption of intermediates under elevated surface temperatures [88,181,182,183]. Such effects are especially pronounced under high-flux or pulsed irradiation, where photon absorption leads to localized thermal gradients.

Hybrid systems that integrate SiC with visible-light-responsive semiconductors improve charge separation and CO_2_ activation. In Cu_2_O/SiC composites, photogenerated electrons in SiC transfer to Cu_2_O, which functions as an electron sink and facilitates CO_2_ reduction to methanol, with yields reaching 191 µmol/g under visible light [184]. Similarly, 3D g-C_3_N_4_/SiC composites utilize SiC whiskers as conductive bridges, achieving CO production rates of 17.78 µmol/g·h due to efficient electron transport and CO_2_ adsorption on g-C_3_N_4_ surfaces [100].

Hydrothermally synthesized Zn_x_Cd_1-x_S-coated SiC nanowires also show enhanced CO_2_ adsorption and charge separation. These structures promote S-scheme charge transfer, supporting high selectivity and catalytic activity.

Rice husk-derived SiC/C composites leverage the inherent conductivity of carbon matrices to improve charge mobility and reduce recombination losses. When modified with small amounts of noble metals (e.g., 5 wt% Pt), these materials display high CO_2_ reduction efficiency under visible light, combining sustainability with performance [79,101].

**Figure 13:**
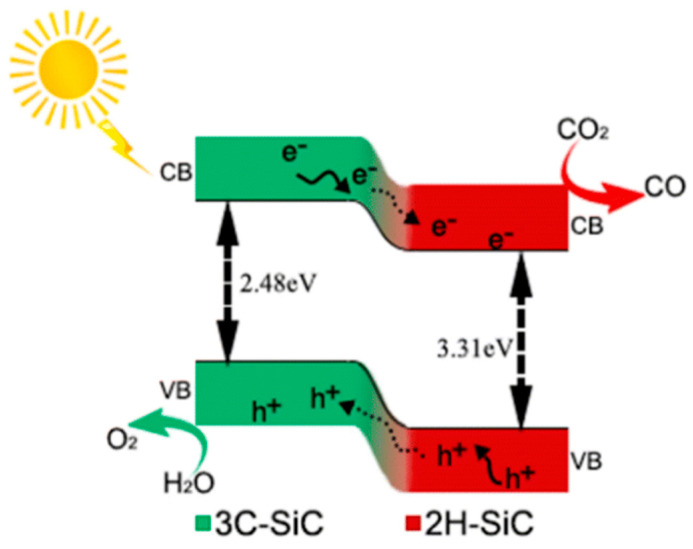
Schematic diagram of the proposed mechanism for photocatalytic reduction of CO_2_ on the 2H–3C SiC catalyst [185].

A particularly effective strategy involves engineering internal phase interfaces within SiC itself. Wang et al. [185] proposed a high-temperature in situ solvothermal synthesis method to fabricate PVP-tuned 2H–3C SiC composite nanosheets. The 2H phase is formed in situ on the 3C SiC lamellar phase, forming a strong two-phase interface. The SiC nanosheets synthesized by the authors [185] with the maximum content of the 2H–3C two-phase interface exhibit the highest activity in the photocatalytic reduction of CO_2_ to CO. It is found that the interface between the SiC phases accelerate the electron transfer, and the conduction band positions for SiC-0 and SiC-0.3 are approximately −1.14 V and −1.0 V, respectively. Band structure analysis shows that 2H–SiC has better electron delocalization with a band gap of 3.31 eV, while for 3C–SiC, this figure is 2.48 eV. Based on theoretical calculations and experimental data, a mechanism for photocatalytic reduction of CO_2_ on the 2H–3C SiC catalyst was proposed (Figure 13). When illuminated, 3C–SiC and 2H–SiC generate electrons and holes [185]. The resulting internal junction facilitates rapid intraparticle charge separation and accelerates surface reactions.

**Figure 14:**
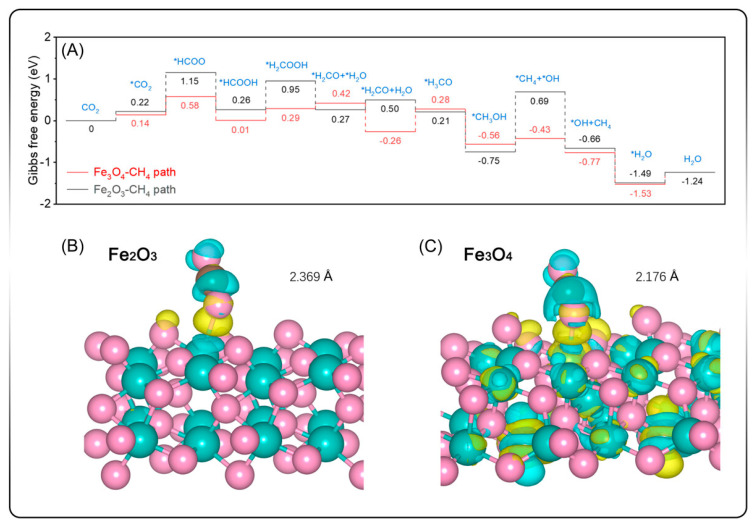
(**A**) Gibbs free energy diagrams for CO_2_-to-CH_4_ pathway over Fe_3_O_4_/SiC and Fe_2_O_3_/SiC. Atomic models with a differential charge density plot of (**B**) Fe_2_O_3_ site and (**C**) Fe_3_O_4_ site. The cyan and yellow regions represent positive and negative charges, respectively, with an isovalue of 0.0025 e^−^/Å^−3^ [186].

Modification of SiC with iron-based cocatalysts allows for selective formation of methane. Lin et al. [186] modified the SiC Fe photocatalyst to reduce CO_2_, with preferential selectivity for CH_4_. In Fe/SiC composites, DFT simulations show that Fe_3_O_4_ offers superior π-backbonding with CO_2_, reducing the activation energy for CH_4_ formation compared with Fe_2_O_3_. The Gibbs free energy for the full eight-electron reduction pathway is more favorable for Fe_3_O_4_ (−0.77 eV) than for Fe_2_O_3_ (−0.66 eV), while CO formation is thermodynamically less favorable, as ΔG is positive (Figure 14A).

The reaction proceeds through *HCOO intermediates, with lower energy barriers than those for *COOH formation. Differential charge density maps confirm stronger CO_2_ binding on Fe_3_O_4_, with shorter bond distances and enhanced electron density at the metal–adsorbate interface (Figure 14B,C). These findings highlight the importance of electronic structure control at the catalyst–adsorbate interface in directing product selectivity [186].

Overall, SiC-based systems for photocatalytic CO_2_ reduction benefit from several complementary design strategies: photothermal activation enhances reaction kinetics and selectivity; heterojunctions (e.g., SiC/Cu_2_O and SiC/g-C_3_N_4_) improve charge separation and surface activation; internal phase engineering (e.g., 2H–3C interfaces) boosts intraparticle charge transport; and cocatalysts (e.g., Fe_3_O_4_) modulate reaction pathways toward desirable products such as CH_4_.

These advances position SiC as a viable platform for solar-to-chemical conversion systems capable of selective CO_2_ utilization. Further work should focus on integrated systems that combine photothermal, electronic, and surface engineering to enable scalable deployment under realistic operating conditions.

## 9. Conclusions and Future Directions

SiC has emerged as a highly promising platform for photocatalytic and photoelectrochemical applications due to its unique combination of physicochemical stability, band gap tunability, and structural versatility. This review has summarized the current state of research on SiC-based materials, encompassing synthesis methods, morphological engineering, doping strategies, heterojunction construction, and their application in water splitting, dye degradation, CO_2_ reduction, and wastewater purification.

The performance of SiC can be significantly improved through nanostructuring, including nanowires, thin films, porous foams, composite membranes, and hybrid architectures. Coupling SiC with cocatalysts, plasmonic metals, and carbon-based conductors further enhances charge-carrier extraction and stability. Advanced heterostructures, particularly Z-scheme and S-scheme systems, effectively suppress recombination while preserving strong redox potentials. Doping strategies (e.g., B, Ce, and Yb) and surface functionalization enable the tuning of band edge positions, photon absorption, and interfacial kinetics.

Computational modeling, including DFT, TD-DFT, GW-BSE, molecular dynamics, and machine learning approaches, plays a critical role in guiding SiC photocatalyst design. These tools provide insights into band structures, carrier transport, interface energetics, and reaction pathways, helping to reduce experimental trial and error and enabling targeted optimization. Integrating theory with experiments will be essential to accelerating future SiC development.

In photocatalytic water splitting, SiC-based heterojunctions such as SiC/PtSe_2_ and CeO_2_/SiC exhibit strong hydrogen evolution performance and, in some cases, high theoretical solar-to-hydrogen (STH) efficiency. In environmental purification, SiC composites achieve robust degradation of dyes, pharmaceuticals, and pesticides under visible light with minimal photocorrosion. For CO_2_ reduction, engineered SiC interfaces (e.g., 2H–3C SiC, SiC/Cu_2_O, and SiC/g-C_3_N_4_) enable selective production of CO, CH_4_, and methanol, demonstrating the material’s versatility across solar–fuel applications.

Despite these advances, several challenges remain. The wide band gap of pristine SiC limits visible-light utilization, requiring complex heterostructure architecture or dopant-induced band engineering. Scalable, low-temperature, and environmentally sustainable synthesis routes are still underdeveloped. Our mechanistic understanding of active sites, radical pathways, interfacial charge dynamics, and long-term stability under realistic operating conditions is incomplete. The design of multi-component systems also demands deeper insights into synergistic interactions and kinetic bottlenecks.

Future research should prioritize the development of tandem photocatalytic systems capable of coupling CO_2_ reduction with water oxidation, enabling complete solar-to-fuel cycles. Machine learning-driven inverse design and high-throughput computational screening are expected to accelerate discovery of optimized SiC compositions and morphologies. The advancement of scalable synthesis using renewable precursors (e.g., agricultural waste) represents a promising direction for sustainable production. Implementing SiC-based catalysts in continuous-flow, photoreactor, and photothermal platforms will be essential to translating laboratory results into industrially relevant processes. Finally, long-term ecological safety, recyclability, and performance under natural sunlight should be systematically evaluated to facilitate real-world deployment.

Overall, SiC-based photocatalysts offer a durable, versatile, and sustainable route toward solar energy conversion and environmental remediation. Through the combined application of advanced synthesis methods, structural engineering, and computational modeling, SiC materials are positioned to play an increasingly central role in next-generation photocatalytic technologies.

## Figures and Tables

**Table 1:** Pure SiC and doped SiC.

Material	Band Gap (eV)	Light Source	Electrolyte	Stability	H_2_ Evolution Rate	Shape/Size	Crystal Structure/Surface Area	Source
SiC nanoparticles	2.7	150 W Xe lamp, AM 1.5	pH adjusted with NaOH/HCl	Decreased over time	36 µmol/g/h (1st h) and 25 µmol/g/h (6 h)	Nanoparticles, 9 nm	β-SiC (3C-SiC)	[85]
Ni-loaded β-SiC nanowires	2.33	300 W Xe lamp, >420 nm	Na_2_S–Na_2_SO3	Stable, 4 cycles	11.1 µL/3 h	Nanowires, ~25 nm	3C-SiC, no impurities	[90]
β-SiC nanowires, acid-modified	2.27–2.35	300 W Xe lamp, >420 nm	Pure water	Stable, 30 h	61 mL/g/h	Nanowires, 70–400 nm	β-SiC, 45 m^2^/g	[158]
Green SiC powder	~2.3	150 W Xe lamp, UV filter	Pure water, Na_2_S, CH_3_OH, and EDTA	Consistent	24.9 µL/g/h	Particles, 400–500 nm	6H-SiC and 3C-SiC	[86]
3C-SiC nanocrystals	2.24	500 W Xe lamp	0.5 M Na_2_SO_4_	Stable photocurrent	H_2_ bubbles observed	Nanocrystals, 1.5–7.5 nm	3C-SiC	[159]

**Table 2:** SiC with metal/non-metal composites.

Material	Band Gap (eV)	Light Source	Electrolyte	Stability	H_2_ Evolution Rate	Shape/Size	Crystal Structure/Surface Area	Doping/QY/AQE	Source
Ag/SiC/g-C_3_N_4_	2.79 (g-C_3_N_4_)	350 W Xe lamp	0.5 M Na_2_SO_4_	Stable, 4 cycles	2971 µmol/h/g	SiC nanofibers, ~25 nm; Ag nanodots, ~10 nm	β-SiC, g-C_3_N_4_, Ag	AQE: 7.3% at 420 nm	[130]
NiO_x_/SiC/CNOs	2.4	300 W Xe lamp, >420 nm	TEOA, Eosin Y	Stable, 3 cycles	3160.2 µmol/g/h	SiC nanowires, 100–200 nm; NiO_x_, 6–10 nm	β-SiC, defect sites	NiO_x_ cocatalyst	[117]
Pt/SiC nanowires	~2.48	300 W Xe lamp	Distilled water	Stable, 20 h	4572 µL/g/h	Nanowires, ~50 nm; Pt, ~2.5 nm	3C-SiC	Pt loading	[81]
Au/SiC	2.4	300 W Xe lamp, >420 nm	0.5 M Na_2_SO_4_	Stable, 4 h	53.6 µmol/h/g	SiC, ~5 µm; Au, 4–5 nm	Hexagonal SiC, 14.7 m^2^/g	Au nanoparticles	[155]
SiC-graphene	2.4	300 W Xe lamp, >420 nm	Distilled water	Stable, 12 h	87.52 µL/g/h	SiC, ~5 µm; graphene sheets	β-SiC, 24 m^2^/g	Graphene bonding	[160]

**Table 3:** SiC with semiconductor composites.

Material	Band Gap (eV)	Light Source	Electrolyte	Stability	H_2_ Evolution Rate	Shape/Size	Crystal Structure/Surface Area	Doping/QY/AQE	Source
MoS_2_/SiC/GO	1.94	Xe lamp, 400–700 nm	1 M Na_2_S and 1 M Na_2_SO_3_	Stable, multiple cycles	4.203 mL/4 h	Sheet-like SiC, MoS_2_, and GO layers	6H-SiC	QY: 21.69% at 400–700 nm	[131]
GO/SiC/MoS_2_	2.61–2.91	Xe lamp, 400–700 nm	0.1 M Na_2_S and 0.1 M Na_2_SO_3_	Stable, 3 cycles	43.59 µmol/h/g	SiC nanosheets, GO, and MoS_2_	6H-SiC, 3.73 m^2^/g	QY: 20.45% at 400–700 nm	[97]
Si/SiC	1.01 (Si) and 2.36 (SiC)	300 W Xe lamp	Deionized water	Stable, >5 h	14.01 µmol/h/g	Rod-like, fibrous, 20–60 nm	3C-SiC and Si, 54.95 m^2^/g	-	[161]
SiC/SnO_2_	2.39 (SiC)	300 W Xe lamp	0.1 M Na_2_S and 0.1 M Na_2_SO_3_	Stable, 14 h	1887.3 µmol/g (4 h)	SiC nanofibers, ~200 nm; SnO_2_ nanosheets, ~10 nm	Cubic SiC and rutile SnO_2_, 28.6 m^2^/g	-	[162]
SiC/CdS/Pt	2.4 (SiC and CdS)	300 W Xe lamp, >420 nm	0.1 M Na_2_S and 0.1 M Na_2_SO_3_	Stable, 12 h	5460 µmol/h/g (with Pt)	Micro-SiC and CdS, ~100 nm	Cubic-hexagonal SiC and cubic CdS, 54 m^2^/g	AQE: 2.1% at 420 nm	[163]
Mesoporous SiC/CdS	1.63	300 W Xe lamp, >420 nm	0.01 M Na_2_S and 0.01 M Na_2_SO_3_	Stable, 16 h	952 µmol/h/g	Worm-like SiC, ~0.3 µm; CdS nanoparticles	Cubic SiC and cubic CdS, 614 m^2^/g	-	[164]
g-C_3_N_4_/SiC	2.7 (g-C_3_N_4_) and 2.4 (SiC)	Visible light, >420 nm	8 mL of TEOA	High stability	182 µmol/g/h	g-C_3_N_4_ sheets, SiC particles	Cubic and hexagonal SiC, 12.52 m^2^/g	-	[165]

## Data Availability

No new data were created or analyzed in this study. Data sharing is not applicable to this article.
